# Metastatic renal carcinoma comprehensive prognostic system

**DOI:** 10.1038/sj.bjc.6600768

**Published:** 2003-02-10

**Authors:** J Atzpodien, P Royston, T Wandert, M Reitz, and DGCIN - German Cooperative Renal Carcinoma Chemo-Immunotherapy Trials Group

**Affiliations:** 1 Medizinische Hochschule Hannover, Carl-Neuberg-Str. 1, 30625 Hannover, Germany; 2 Fachklinik Hornheide an der Universität Münster, Dorbaumstr. 300, 48157 Münster, Germany; 3 Europäisches Institut für Tumor Immunologie und Prävention (EUTIP), Gotenstr. 152, 53175 Bonn, Germany; 4 Medical Research Council (MRC), Cancer Division, MRC Clinical Trials Unit, 222 Euston Road, London NW1 2DA, UK

**Keywords:** renal-cell carcinoma, immunotherapy, survival, prognosis, risk, stratification

## Abstract

The purpose of the study was to identify a comprehensive prognostic system of pretreatment clinical parameters in 425 patients (pts) with metastatic renal-cell carcinoma treated with different subcutaneous (s.c.) recombinant cytokine-based home therapies in consecutive trials. Treatment consisted of (A) s.c. interferon-*α*2a (INF-*α*), s.c. interleukin-2 (IL-2) (*n*=102 pts), (B) s.c. IFN-*α*2a, s.c. IL-2, and i.v. 5-fluorouracil (5-FU) (*n*=235 pts) or (C) s.c. IFN-*α*2a, s.c. IL-2, and i.v. 5-FU combined with p.o. 13-*cis*-retinoic acid (13cRA) (*n*=88 pts). Kaplan–Meier survival analysis, log-rank statistics, and Cox regression analysis were employed to identify risk factors and to create a multiple risk factor model. The following pretreatment risk factors were identified by univariate analysis: (1) three and more metastatic sites, (2) presence of liver, lymph node or bone metastases, (3) neutrophil count ⩾6500 cells *μ*l^−1^, (4) serum lactate dehydrogenase level (LDH) ⩾220 U l^−1^, and (5) serum C-reactive protein level (CRP) ⩾11 mg l^−1^. Cox regression analysis with forward stepwise variable selection identified neutrophil count as the major prognostic factor (hazard ratio=1.9, *P*<0.001), while serum levels of LDH and CRP, time between diagnosis of tumour and onset of metastatic disease, number of metastatic sites, and bone metastases were significant but somewhat less important prognostic variables within the multiple risk factor model (hazard ratio ⩽1.5). Patients were assigned to one of the three risk groups according to cumulative risk defined as the sum of simplified risk s.c.ores for six pretreatment variables. Low-, intermediate-, and high-risk patients achieved a median overall survival of 32+ months (95% CI 24, 43; 5-year survival of 27%), 18+ months (95% CI 15, 20; 5-year survival of 11%), and 8+ months (95% CI 6, 10; 5-year survival of 5%), respectively. These prognostic categories are helpful both in individual patient care and in the assessment of patients entering prospective clinical trials.

Patients (pts) with untreated metastatic renal-cell carcinoma have an overall median survival of no more than 12 months and a 5-year survival of less than 10%. While renal-cell carcinoma responds poorly to single-agent chemotherapy or hormonal therapy, immunotherapies with subcutaneous (s.c.) recombinant interleukin-2 (IL-2) alone or in combination with s.c. recombinant interferon-*α* (INF-*α*) yielded significant therapeutic efficacy in renal-cell carcinoma (Atzpodien *et al*, 1990,^2001^,^2002^; Sleijfer *et al*, 1992).

In previous studies, a variety of prognostic staging factors, notably, performance status, recent weight loss, disease-free interval, pretreatment erythrocyte sedimentation rate (ESR), lactate dehydrogenase (LDH), neutrophils, haemoglobin, extrapulmonary and bone metastases, and a number of metastatic sites were identified as important indicators for survival in metastatic renal-cell carcinoma patients (Elson *et al*, 1988; Palmer *et al*, 1992; Lopez-Hänninen *et al*, 1996; Gelb, 1997; Culine *et al*, 1998; Hoffmann *et al*, 1999; Motzer *et al*, 1999,^2002^).

Here, we develop a comprehensive new prognostic system for metastatic renal carcinoma patients, by retrospective analysis. All patients were treated with outpatient immunotherapy comprising s.c. IL-2 and s.c. INF-*α*.

## PATIENTS AND METHODS

### Patients

Between November 1988 and February 1998, 425 patients with progressive metastatic renal-cell carcinoma were entered on consecutive clinical trials and received either IFN-*α*2a, IL-2 (therapy A, *n*=102 pts), IFN-*α*2a, IL-2, and 5-FU (therapy B, *n*=235 pts) or IFN-*α*2a, IL-2, and 5-FU combined with 13cRA (therapy C, *n*=88 pts). Median follow-up of these patients was 20+ months (range 0–157+ months). Pretreatment included radical tumour nephrectomy (*n*=412), chemotherapy (*n*=5), immunotherapy (*n*=47), chemoimmunotherapy (*n*=8), and hormone therapy (*n*=32).

Since all treatment regimens were designed to be administrated at home, selection of patients with good or excellent performance status was required. Criteria for entry into the study were: histologically confirmed metastatic renal-cell carcinoma; an expected survival duration of more than 3 months; Karnofsky performance status >80%; age between 18 and 80 years; white blood cell count >3500 *μ*l^−1^; platelet count >100 000 *μ*l^−1^; haematocrit >30%; serum bilirubin and creatinin <1.25 of the upper normal limit. Exclusion criteria included evidence of congestive heart failure, severe coronary artery disease, cardiac arrhythmias, symptomatic CNS disease or seizure disorders, human immunodeficiency virus infections or positivity for hepatitis B surface antigen or chronic hepatitis, or concomitant corticosteroid therapy. In all patients treated, no chemotherapy, immunomodulatory treatment, or steroid therapy had been performed during the previous 4 weeks. Pregnant and lactating woman were excluded.

The clinical studies were approved by the institutional review board of the Medizinische Hochschule Hannover; written informed consent was obtained from all patients prior to entry into the study.

### Treatment design

Patients were treated in an outpatient setting. All patients received outpatient s.c. IFN-*α*2a and s.c. IL-2. Treatment A consisted of IFN-*α*2a and IL-2, only. s.c. IFN-*α*2a was administered at 5×10^6^ IU m^−2^, day 1, weeks 1+4, and days 1, 3, 5, weeks 2, 3, 5, 6; s.c. IL-2 was administered at 10×10^6^ IU m^−2^, twice daily days 3–5 weeks 1+4, and at 5×10^6^ IU m^−2^, days 1, 3, 5, weeks 2, 3, 5, 6; weeks 7 and 8 were therapy free. Treatment B consisted of IFN-*α*2a, IL-2, and 5-FU. s.c. IFN-*α*2a was administered at 5×10^6^ IU m^−2^, day 1, weeks 1+4, and days 1, 3, 5, weeks 2+3, and at 10×10^6^ IU m^−2^, days 1, 3, 5, weeks 5–8. s.c. IL-2 was administered at 10×10^6^ IU m^−2^, twice daily days 3–5 weeks 1+4, and at 5×10^6^ IU m^−2^, days 1, 3, 5, weeks 2+3; i.v. bolus 5-FU was administered at 1000 mg m^−2^, day 1 weeks 5–8. Treatment C consisted of IFN-*α*2a, IL-2, 5-FU, and 13-cRA; patients received 20 mg p.o. 13cRA twice daily, in addition to the above dosages of IFN-*α*2a, IL-2, and 5-FU.

Eight week treatment cycles were repeated for up to three courses unless progression of disease occurred. Re-evaluation of patients' tumour status was performed between treatment cycles.

Concomitant medication was given as needed to control adverse effects of chemoimmuno-therapy.

### Statistical analysis

Survival was measured from start of therapy to date of death or to the last date known to be alive. Treatment efficacy was assessed on an intention-to-treat basis. Survival curves were estimated by the Kaplan–Meier method. Univariate risk factor analysis was performed by the log-rank test and multivariate analysis by Cox regression. Continuous pretreatment clinical measurements (e.g. neutrophil count) were analysed as dichotomous variables according to approximately ‘optimal’ cutpoints, determined as follows. The value best discriminating between good and poor survival (i.e., which had the most significant *P*-value on a log-rank test) was found by testing all possible cutpoints within the central 80% of the distribution of values. All such cutpoints were then rounded to clinically relevant (i.e. convenient) values. The *P*-values for the clinically relevant cutpoints were corrected for multiple testing (Miller and Siegmund, 1982; Altman *et al*, 1994). Cox regression analysis with forward stepwise variable selection was employed to build a model with multiple risk factors. To allow for possible joint effects, all predictors were included as candidate variables, whether or not significant in univariate analysis.

## RESULTS

**Figure 3:**
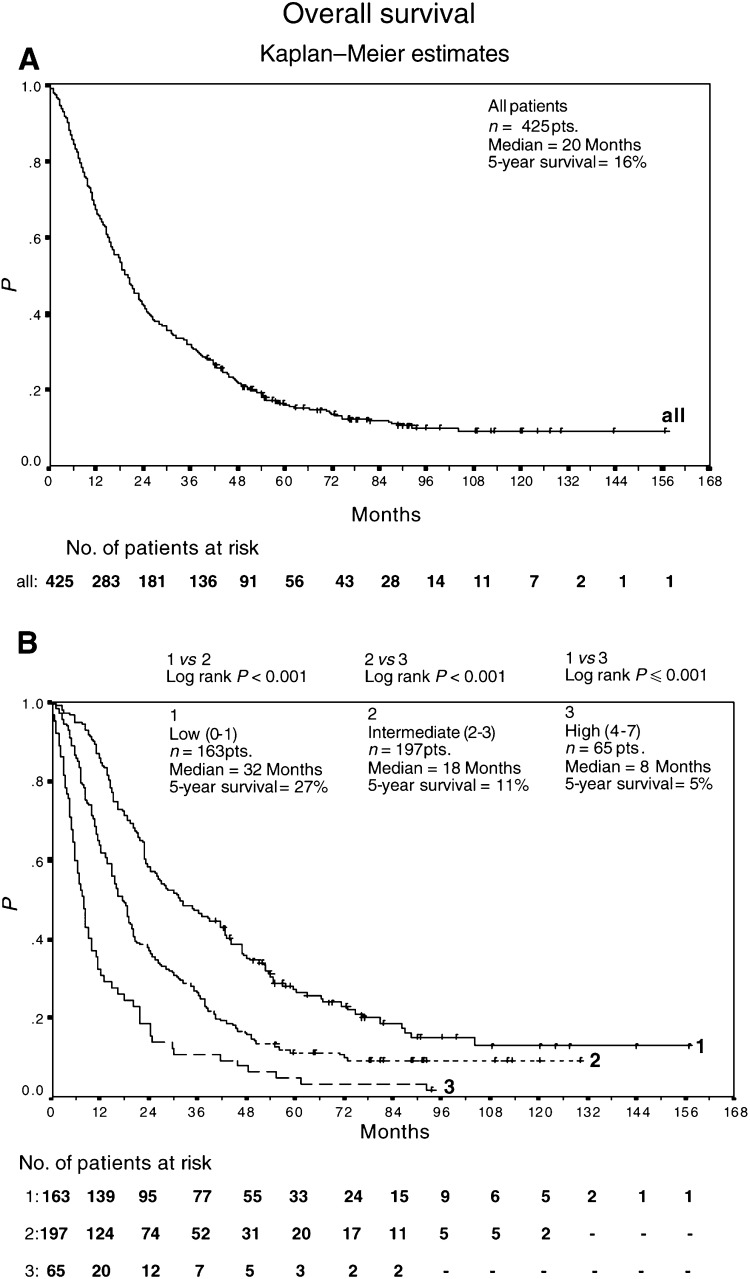
Overall survival of 425 advanced renal-cell carcinoma patients treated with outpatient s.c. IL-2/INF-*α*2a therapy (**A**). Overall survival of 163 low-risk patients, 197 intermediate-risk patients, and 65 high-risk patients treated with outpatient subcutaneous interleukin-2/interferon-*α*2a therapy (**B**). Survival was calculated from the start of therapy using Kaplan–Meier method.

Median survival time was 20+ months (95% CI, 18, 22; 5-year survival of 16%) (Figure 3A

); 54 of 425 patients remain alive.

### Univariate risk factor analysis

As shown in Table 1

**Table 1:** Pretreatment clinical factors and their prognostic significance in univariate analysis

**Risk factors^a^**	**Categories compared**	**Number of patients**	**Median survival (months)**	***P*-value (log-rank test)**	***P*-value (corrected^b^)**
Sex	Female *vs* male	114 *vs* 311	20 *vs* 19	0.5	
Age (years)	< 50 *vs* ⩾50	80 *vs* 345	13 *vs* 21	0.06	0.5
Pretreatment with IL-2	Absent *vs* present	370 *vs* 55	20 *vs* 17	0.6	
Time from diagnosis of tumour to metastatic disease (years)	< 3 *vs* ⩾3	304 *vs* 121	17 *vs* 25	0.01	0.2
Number of metastatic sites	< 3 *vs* ⩾3	350 *vs* 75	21 *vs* 14	<0.001	0.003
Lung metastases	Absent *vs* present	113 *vs* 312	15 *vs* 21	0.2	
Liver metastases	Absent *vs* present	362 *vs* 63	20 *vs* 15	0.03	
Lymph node metastases	Absent *vs* present	302 *vs* 123	21 *vs* 15	0.02	
Brain metastases	Absent *vs* present	400 *vs* 25	20 *vs* 20	0.2	
Bone metastases	Absent *vs* present	337 *vs* 88	22 *vs* 12	<0.001	
Other metastases	Absent *vs* present	284 *vs* 141	21 *vs* 18	0.2	
					
ESR (mm h^−1^)^c^	< 60 *vs* ⩾60	343 *vs* 82	22 *vs* 11	0.002	0.05
Hemoglobin (g dl^−1^)^c^	< 11 *vs* ⩾11	48 *vs* 377	10 *vs* 21	0.06	0.53
Neutrophil counts (cells *μ*l^−1^)^c^	< 6500 *vs* ⩾6500	362 *vs* 63	21 *vs* 8	<0.001	<0.001
LDH (U l^−1^)^c^	< 220 *vs* ⩾220	330 *vs* 95	22 *vs* 12	<0.001	0.01
CRP (mg l^−1^)^c^	< 11 *vs* ⩾11	222 *vs* 203	24 *vs* 16	<0.001	<0.001

a Laboratory normal ranges were as follows: ESR: male: 3–8 mm h^−1^, female: 6–11 mm h^−1^; haemoglobin: male: 13.5–17.5 g dl^−1^, female: 12–16 g dl^−1^; neutrophils: 1500–7500 cells *μ*l^−1^; LDH: 80–240 U l^−1^; CRP: <5 mg l^−1^.

b Corrected for testing multiple cutpoints on continuous factors (Miller and Siegmund, 1982).

c Cutoff does not reflect normal range.

, we identified the following pretreatment staging factors as univariate predictors of poor overall survival: (1) three and more metastatic sites, (2) presence of liver, lymph node or bone metastases, (3) neutrophil counts ⩾6500 cells *μ*l^−1^, (4) serum LDH level ⩾220 U l^−1^, and (5) serum C-reactive protein level (CRP) ⩾11 mg l^−1^.

Sex, age, time from diagnosis of tumour to metastatic disease, the presence of lung, brain or other metastases, ESR, haemoglobin level, and IL-2-pretreatment were also tested, but rendered not significant by univariate analysis after correction (where necessary) of *P*-values by using the formula of Miller and Siegmund (1982) (see also Altman *et al*, 1994).

### Multivariate analysis of risk factors and overall survival

To build a multiple risk factor model, we used multivariate Cox regression containing all predictors as candidate variables, since factors that are not univariately significant may nevertheless become significant when included together in the model. Six factors were found to be significant in a multivariate fashion. Neutrophil count was identified as the major prognostic factor (hazard ratio=1.9, *P*<0.001), while serum level of LDH (hazard ratio 1.3; *P*=0.02) and CRP (hazard ratio 1.4; *P*=0.001), time between diagnosis of tumour and metastatic disease (hazard ratio 0.7; *P*=0.001), number of metastatic sites (hazard ratio 1.4; *P*=0.01), and bone metastases (hazard ratio 1.5; *P*=0.001) were significant but less important prognostic variables within the multiple risk factor model (Table 2

**Table 2:** Multivariate risk factor model for overall survival in metastatic renal carcinoma

**Risk factor**	**Categories compared**	**Hazard ratio^a^**	**95% CI^a^**	***P*-value^a^**	**Weight (contribution to cumulative risk score)**
Neutrophil counts (cells *μ*l^−1^)	<6500 *vs* ⩾6500	1.9	1.5–2.6	<0.001	0 *vs* 2
LDH (U l^−1^)	<220 *vs* ⩾220	1.3	1.0–1.7	0.02	0 *vs* 1
CRP (mg l^−1^)^a^	<11 *vs* ⩾11	1.4	1.1–1.7	0.001	0 *vs* 1
Time from diagnosis of tumour to metastatic disease (years)	<3 *vs* ⩾3	0.7	0.5–0.9	0.001	1 *vs* 0
Number of metastatic sites	<3 *vs* ⩾3	1.4	1.1–1.9	0.01	0 *vs* 1
Bone metastases	Absent *vs* present	1.5	1.2–2.0	0.001	0 *vs* 1

a In multivariate Cox regression model.

). However, caution should be exercised regarding the significance of these *P*-values since methodology seems to be unavailable to correct for the selection of ‘optimal’ cutpoints within a multivariate modelling framework.

**Figure 1:**
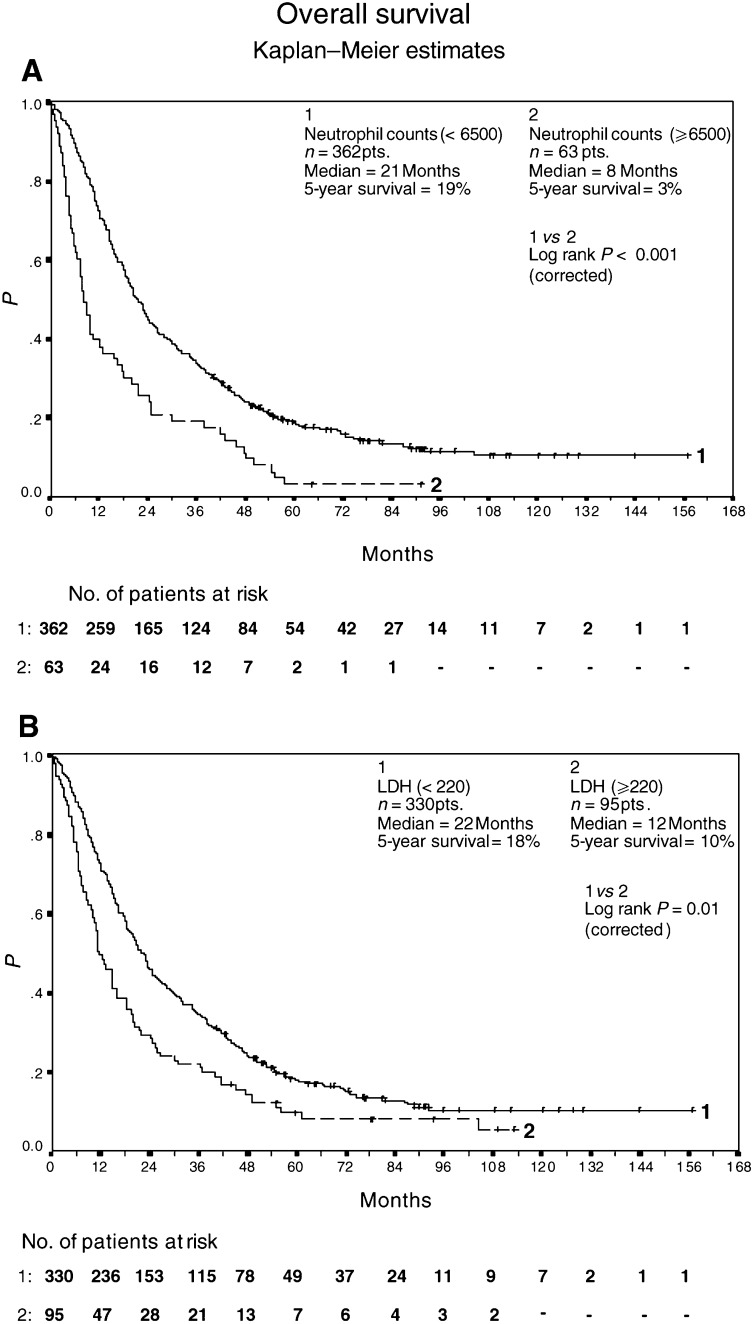
Overall survival of 362 patients with neutrophil counts <6500 cells *μ*l^−1^ and 63 patients with neutrophil counts ⩾6500 cells *μ*l^−1^ (**A**). Overall survival of 330 patients with LDH levels <220 U l^−1^ and 95 patients with LDH levels ⩾220 U l^−1^ (**B**). All patients were treated with outpatient s.c. IL-2/IL-*α*2a therapy. Survival was calculated from the start of therapy using Kaplan–Meier method.

The largest and most significant association with an unfavourable outcome was observed in patients with elevated neutrophil count (⩾6500 cells *μ*l^−1^) (Figure 1A

). A total of 63 patients with elevated levels of neutrophils count achieved a median overall survival of 8+ months (95% CI 6, 12; 5-year survival of 3%), while 362 patients with less than 6500 cells *μ*l^−1^ yielded a median overall survival of 21+ months (95% CI 19, 24; 5-year survival of 19%). Similarly, 95 patients with elevated LDH levels yielded a median overall survival of 12+ months (95% CI 10, 16; 5-year survival of 10%), in contrast to 330 patients with LDH levels less than 220 U l^−1^, and a median overall survival of 22+ months (95% CI 19, 25; 5-year survival of 18%).

**Figure 2:**
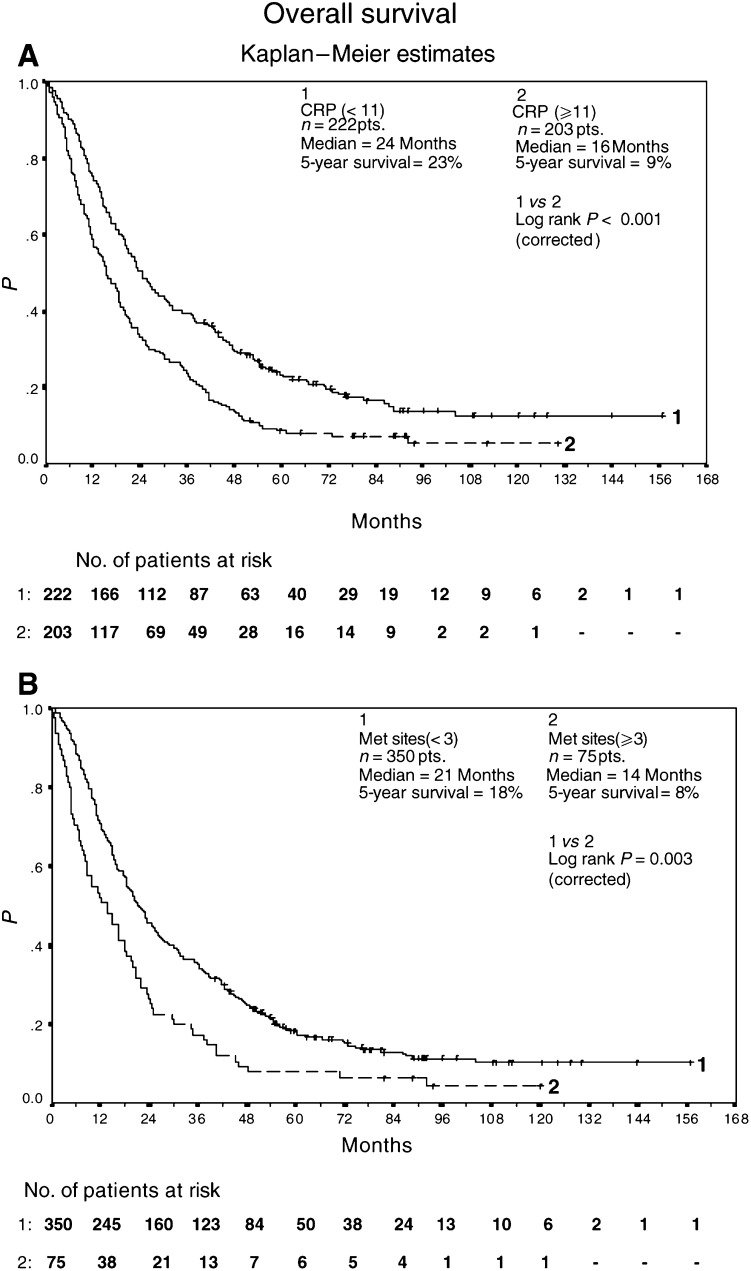
Overall survival of 222 patients with CRP levels <11 mg l^−1^ and 203 patients with CRP levels ⩾11 mg l^−1^ (**A**). Overall survival of 350 patients with <3 metastatic sites and 75 patients with ⩾3 metastatic sites (**B**). All patients were treated with outpatient s.c. IL-2/INF-*α*2a therapy. Survival was calculated from the start of therapy using Kaplan–Meier method.

It was calculated that 203 patients with elevated serum levels of CRP achieved a median overall survival of 16+ months (95% CI 12, 19; 5-year survival of 9%), while 222 patients with CRP levels less than 11 mg l^−1^ yielded a median overall survival of 24+ months (95% CI 21, 30; 5-year survival of 23%) (Figure 2A

). In addition, 75 patients with three and more metastatic sites had a median overall survival of 14+ months (95% CI 8, 18; 5-year survival of 8%), compared to 350 patients with one or two metastatic sites and a median overall survival of 21+ months (95% CI 19, 25; 5-year survival of 18%) (Figure 2B).

### Prognostic system

Based on the rounded regression coefficients (log hazard ratios in the final Cox model) of variables, we defined the weights of prognostic features as follows: neutrophil count was assigned weight 2, the remaining variables (serum level of CRP and LDH, time between diagnosis of tumour and metastatic disease, number of metastatic sites, bone metastases) were given weight 1. A prognostic score consisting of the sum of the weights of these six variables was used to assign patients to low (0⩾score⩽1), intermediate (2⩾score⩽3), and high risk (4⩾score⩽7) groups, respectively (Table 3

**Table 3:** Definition of risk groups from cumulative risk score

**Risk group**	**Cumulative risk score**	**Contribution of individual prognostic variables**
Low risk (*n*=163)	0, 1	Absence
		One minor prognostic variable^a^
Intermediate (*n*=197)	2, 3	Two minor prognostic variables
		Three minor prognostic variables
		One minor plus major prognostic variable^b^
High risk (*n*=65)	4, 5, 6, 7	Four or five minor prognostic variables
		⩾Three minor plus major prognostic variable

a i.e. with weight 1.

b i.e. with weight 2.

).

Median overall survival of low (*n*=163), intermediate (*n*=197), and high-risk (*n*=65) patients was 32+ months (95% CI 24, 43; 5-year survival of 27%), 18+ months (95% CI 15, 20; 5-year survival of 11%), and 8+ months (95% CI 6, 10; 5-year survival of 5%), respectively (Figure 3B).

## DISCUSSION

The objective of this study was to devise a comprehensive new prognostic system for survival of metastatic renal carcinoma patients.

Using a multivariate risk model derived from the retrospective analysis of 425 patients with metastatic renal-cell carcinoma, we categorised patients into three distinct risk groups based on the following six prognostic factors for poor survival: (1) neutrophil count ⩾6500 cells *μ*l^1^, (2) serum level of LDH ⩾220 U l^−1^, (3) serum level of CRP ⩾11 mg l^−1^, (4) time between diagnosis of tumour and metastatic disease less than 3 years, (5) three and more metastatic sites, and (6) the presence of bone metastases.

These prognostic variables in advanced renal cancer were comparable to clinical features reported previously by others, notably with regard to the number of metastatic sites (Elson *et al*, 1988), bone metastases (Mani *et al*, 1995), time between diagnosis of tumour and metastatic disease (Maldazys and deKernion, 1986; Elson *et al*, 1988; Motzer *et al*, 2002), and serum level of LDH (Motzer *et al*, 1999,^2002^).

In this current and in our previous study (Lopez-Hänninen *et al*, 1996), for the first time, we could identify pretreatment neutrophil count as a highly statistically significant predictor for overall survival in advanced renal-cell carcinoma. While the biological interpretation of increased neutrophil counts is not evident, Blay *et al* (1997) demonstrated that IL-6 associated neutrophilia in renal-cell carcinoma could be decreased via the suppression of IL-6 or IL-6-associated paraneoplastic inflammatory syndrome in renal carcinoma. Notably, Paesmans *et al* (1995),(2000) and Kapp *et al* (1983) also showed its impact as a prognostic factor for survival in small-cell lung cancer and uterine cervix carcinoma, respectively.

Surprisingly, in this report the presence of brain or CNS metastases (*n*=25) had no selective impact on survival, which may have been because of concomitant extensive multiorgan disease. Alternatively, the small number of such patients may have reduced the statistical power to detect such an impact in the presence of other important risk factors.

Similar to Motzer *et al* (2002), who categorised IFN-*α*-treated advanced renal-cell cancer patients into three different groups with low (18%; overall survival 30 months), intermediate (62%; overall survival 14 months), and high-risk patients (20%; overall survival 5 months), respectively, we established three distinct survival subgroups that is, low-risk patients (38%) with an overall median survival of 32 months, intermediate-risk patients (47%) with an overall median survival of 18 months, and high-risk patients (15%) with an overall median survival of 8 months. Notably, while Motzer *et al* (2002) also included LDH, in our current model, performance status and serum calcium were not tested, and serum haemoglobin was not identified as a significant statistical predictor for overall survival. In the present prognostic model, risk groups exhibited well-separated survival curves that reflected the prognosis of good/excellent performance status of metastatic renal-cell carcinoma patients receiving outpatient IL-2/IFN-*α*2a. Overall, this group was highly selected as demonstrated by the relatively large number of patients who had a delay between primary diagnosis and metastatic disease in excess of 3 years.

The identification of prognostic features for overall survival in metastatic renal carcinoma patients has a pivotal role in defining future individualised molecular treatment approaches. The low proportion of patients achieving long-term survival suggest the need for further clinical trials of new therapeutic agents.

While there is a partial consensus between different prognostic models in metastatic renal-cell carcinoma, validation of our proposed model will require testing in a prospectively designed study.
